# The Independent Practitioner

**Published:** 1883-04

**Authors:** 


### "THE INDEPENDENT PRACTITIONER."

We are under obligations to a number of our dental contemporaries for unsolicited favorable notices, acts of kindness and appreciation which we shall not soon forget. We hope to present much more matter which shall meet the approbation of those most competent judges. Besides other valuable articles, we have now in hand two of unusual interest, from the pen of that most indefatigable student, Dr. W. D. Miller, of Berlin, and an apology is due, both to the author and our readers, for keeping them back so long. But emergencies which every editor will readily comprehend have conspired to prevent their early appearance. They will not spoil by keeping, and no judicious host desires to bring out all his dainties at the beginning of a feast. The next issue of THE INDEPENDENT PRACTITIONER will contain at least one of them. It is no disparagement to other observers to say that in the study of the etiology of dental caries, Dr. Miller has made discoveries which entitle him to a first place among dental pathologists.

The present number contains an article by Prof. Frank Abbott, to which we wish to call special attention. He has made an exhaustive study of his theme, is known to the whole dental world as a keen observer and lucid reasoner, and this paper is one of the results of his long-continued researches.

